# Constitutively active MyD88/CD40 costimulation enhances expansion and efficacy of chimeric antigen receptor T cells targeting hematological malignancies

**DOI:** 10.1038/s41375-019-0417-9

**Published:** 2019-02-28

**Authors:** Matthew R. Collinson-Pautz, Wei-Chun Chang, An Lu, Mariam Khalil, Jeannette W. Crisostomo, Pei-Yi Lin, Aruna Mahendravada, Nicholas P. Shinners, Mary E. Brandt, Ming Zhang, MyLinh Duong, J. Henri Bayle, Kevin M. Slawin, David M. Spencer, Aaron E. Foster

**Affiliations:** Bellicum Pharmaceuticals, 2130 W. Holcombe Boulevard Suite 800, Houston, TX 77030 USA

**Keywords:** Cancer immunotherapy, Immunotherapy

## Abstract

Successful adoptive chimeric antigen receptor (CAR) T-cell therapies against hematological malignancies require CAR-T expansion and durable persistence following infusion. Balancing increased CAR-T potency with safety, including severe cytokine-release syndrome (sCRS) and neurotoxicity, warrants inclusion of safety mechanisms to control in vivo CAR-T activity. Here, we describe a novel CAR-T cell platform that utilizes expression of the toll-like receptor (TLR) adaptor molecule, MyD88, and tumor-necrosis factor family member, CD40 (MC), tethered to the CAR molecule through an intentionally inefficient 2A linker system, providing a constitutive signal that drives CAR-T survival, proliferation, and antitumor activity against CD19^+^ and CD123^+^ hematological cancers. Robust activity of MC-enhanced CAR-T cells was associated with cachexia in animal models that corresponded with high levels of human cytokine production. However, toxicity could be successfully resolved by using the inducible caspase-9 (iC9) safety switch to reduce serum cytokines, by administration of a neutralizing antibody against TNF-α, or by selecting “low” cytokine-producing CD8^+^ T cells, without loss of antitumor activity. Interestingly, high basal activity was essential for in vivo CAR-T expansion. This study shows that co-opting novel signaling elements (i.e., MyD88 and CD40) and development of a unique CAR-T architecture can drive T-cell proliferation in vivo to enhance CAR-T therapies.

## Introduction

Adoptive transfer of T cells expressing chimeric antigen receptors (CARs) is an effective therapy for the treatment of certain hematological malignancies [1]. In these patients, antitumor activity is associated with robust CAR-T cell expansion post-infusion that is often associated with toxicity (i.e., severe cytokine-release syndrome (CRS) and neurotoxicity), while patients with poor CAR-T proliferation and persistence show reduced durable remission rates [2–4]. Here, we demonstrate that constitutively active signaling from nonconventional costimulatory molecules, MyD88 and CD40 (MC), can enhance CAR-T survival, proliferative capacity, and antitumor activity. Importantly, the occurrence of cytokine-related toxicity from these highly active CAR-T cells can be controlled using inducible caspase-9 (iC9) to safely maximize tumor killing.

We recently reported that an inducible MyD88/CD40 (iMC) expressed as a ligand-dependent costimulatory molecule could be paired with a first-generation CAR to produce controllable CAR-T cells, requiring systemic administration of the small-molecule dimerizing agent, rimiducid (formerly AP1903), to stimulate T-cell proliferation and antitumor activity [5]. These studies also suggested that the MyD88/CD40 fusion is distinct from conventional costimulatory domains (i.e., CD28, 4-1BB, and OX40) expressed in cis in other CAR-T platforms and greatly enhanced CAR-T cell proliferation. Moreover, Mata et al. demonstrated that iMC-enhanced Her2-targeted CAR-T cells outperformed a CD28 Her2-specific CAR-T construct, which is currently being evaluated in several clinical studies targeting solid tumors [6].

While iMC appears to be a potent costimulatory molecule for driving CAR-T cell proliferation, it relies on activation via rimiducid dimerization of the high-affinity variant FKBP12v36 binding domain, obviating the availability of this ligand for use in rimiducid-mediated iC9 activation [7–9]. To benefit from the costimulatory effects of MC signaling in T cells while preserving the option for rimiducid-dependent activation of the iC9 safety switch, we developed an approach to safely express MC as a constitutively active protein to enhance the proliferation and antitumor activity of CAR-T cells targeting lymphoma and leukemia-specific antigens.

## Materials and methods

### Mice

NOD.Cg-Prkdc^scid^ Il2rg^tm1Wjl^/SzJ (NSG) mice were obtained from Jackson Laboratories (Bar Harbor, ME) and maintained at the Bellicum Pharmaceuticals vivarium. All procedures in this protocol are in compliance with the Animal Welfare Act and the Guide for the Care and Use of Laboratory Animals. These studies were approved by the Bellicum Pharmaceuticals’ Institutional Animal Care and Use Committee (IACUC), comprising both internal and external reviewers from Baylor College of Medicine and the University of Texas Health Sciences. Animal studies were not randomized. All animal studies were conducted unblinded, which was deemed acceptable since no subjective observational data were reported. Sample sizes were chosen to minimize the number of animals required in the reported studies and based on estimates of effect size and variance between treatment groups.

### Cell lines, media, and reagents

293T (HEK 293 T/17), Raji, Daudi, and THP-1 cell lines were obtained from the American Type Culture Collection. Cell lines were maintained in DMEM (Invitrogen, Grand Island, NY) supplemented with 10% fetal calf serum (FCS) and 2 mM GlutaMAX™ (Invitrogen) at 37 °C and 5% CO_2_. T cells generated from peripheral blood mononuclear cells (PBMC) were cultured in 45% RPMI 1640, 45% Click’s media (Invitrogen) supplemented with 10% fetal bovine serum (FBS), 2 mM GlutaMAX (T-cell media, TCM), and 100 U/ml IL-2 (Miltenyi Biotec, Bergisch Gladbach, Germany), unless otherwise noted. Clinical-grade rimiducid (5 mg/ml in 25% Kolliphor HS15^®^) was diluted in ethanol to a 100 mM working solution for in vitro assays, or 0.9% saline for animal studies.

### Retroviral and plasmid constructs

Initial bicistronic SFG-based retroviral vectors were generated encoding iC9 together with a first-generation anti-CD19 CAR, comprising the FMC63 single-chain variable fragment (scFv), the CD8α stalk and transmembrane domain, and the CD3ζ chain cytoplasmic domain (iC9-CD19.ζ). In all CAR vectors, the CD34 Qbend-10 minimal epitope [10] was included in the CD8α stalk to detect CAR expression on gene-modified T cells. We also constructed a third-generation CAR, which included the MC costimulatory proteins proximal to the CD8α transmembrane region (iC9-CD19.MC.ζ). In addition, vectors were constructed with only MyD88 (M) or CD40 (C) for both the third-generation (iC9-CD19.M.ζ and iC9-CD19.C.ζ, respectively). We subsequently built a tricistronic iC9-enabled CD19 and CD123 (26292 scFv [11, 12]) CAR construct with a constitutively expressed MC chimeric protein (iC9-CD19.ζ-MC). iC9-expressing CD19 vectors were also synthesized encoding the CD28 and 4-1BB endodomains, as previously described [13, 14]. Additional vectors were synthesized with enhanced 2A sequences, including GSG linkers to improve ribosomal skipping efficiency [15], as well as alternative orientations of the above transgenes.

For coculture assays and in vivo studies, tumor cell lines were modified with retroviral vectors encoding EGFP*luciferase* (EGFPluc). In some experiments, T cells were labeled with retroviral vector encoding Orange Nano-Lantern (ONL) containing Renilla Luciferase to enable in vivo bioluminescent imaging to track T cells.

### Generation of gene-modified T cells

Retroviral supernatants were produced by transient co-transfection of 293T cells with the SFG vector plasmid, pEQ-PAM3(-E) plasmid containing the sequence for MoMLV gag-pol, and an RD114 envelope-encoding plasmid, using GeneJuice (EMD Biosciences, Gibbstown, NJ) transfection reagent. Activated T cells were made from peripheral blood mononuclear cells (PBMCs) obtained from the Gulf Coast Blood Bank (Houston, TX) and activated using anti-CD3/anti-CD28 antibodies, as previously described [5]. After 3 days of activation, T cells were subsequently transduced on retronectin-coated plates (Takara Bio, Otsu, Shiga, Japan) and expanded with 100 U/ml IL-2 for 10–14 days. For two transductions, the protocol was identical to the above except that the wells were coated with equal amounts of each retroviral supernatant.

### Immunophenotyping

Gene-modified T cells were analyzed for transgene expression 10–14 days post-transduction by flow cytometry using CD3-PerCP.Cy5 (Biolegend Cat:317336) and CD34-PE or APC (Abnova Cat:MAB6483, R&D Systems Cat:FAB7227A). Experiments evaluating cell selection of CAR-T cell subsets (i.e., CD4 and CD8) were tested for purity using CD4 (Cat:344604) and CD8 (Cat:301048) antibodies (BioLegend). Additional phenotypic analyses were conducted using antibodies for CD45RA (Cat:304126) and CD62L (Cat:304810) (T-cell memory phenotype), and PD-1 (T-cell exhaustion, Cat:329920) (Biolegend). All flow cytometry was performed using a Gallios flow cytometer, and the data were analyzed using Kaluza software (Beckman Coulter, Brea, CA).

### Coculture assays

Non-transduced (NT) and gene-modified T cells were cultured at a 1:1 effector-to-target ratio (5 × 10^5^ cells each in a 24-well plate) with CD19^+^ Raji-EGFPluc tumor cells for 7 days in the absence of exogenous IL-2. Cells were then harvested, enumerated, and analyzed by flow cytometry for the frequency of T cells (CD3^+^) or tumor cells (EGFPluc^+^). In some assays, NT and gene-modified T cells were cultured without target cells (5 × 10^5^ cells each in a 24-well plate). Culture supernatants were analyzed for cytokine levels at 48 h after the start of the coculture.

### Animal models

To evaluate antitumor activity of CD19-targeted CAR-T cells, NSG mice were engrafted with 5 × 10^5^ CD19^+^ Raji or Raji-EGFPluc tumor cells by intravenous (i.v.) tail vein injection. After 4 days, variable doses of NT and gene-modified T cells were administered by i.v. (tail) injection. In some experiments, mice were rechallenged with Raji-EGFPluc tumor cells as above. To test CD123-specific CAR-T activity, 1 × 10^6^ CD123^+^ THP-1-EGFPluc tumor cells were engrafted by i.v. injection, followed by infusion of 2.5 × 10^6^ unmodified or CAR-T cells 7 days post-tumor engraftment. iC9 titration experiments were performed by treating Raji tumor-bearing mice with 5 × 10^6^ iC9-CD19.ζ-MC-modified T cells followed by injection of rimiducid 7 days after T-cell injection at 0.00005, 0.0005, 0.005, 0.05, 0.5, and 5 mg/kg. To evaluate cytokine-related toxicities, neutralizing antibodies against hIL-6, hIFN-γ, and TNF-α or an isotype control antibody (Bio X Cell, West Lebanon, NH) were administered by i.p. injection at 100 μg twice weekly. Additional experiments were performed using positively selected CD4^+^ and CD8^+^ iC9-CD19.ζ-MC-modified T cells using CD4 or CD8 microbeads and MACS columns (Miltenyi Biotec). In vivo tumor growth and T-cell proliferation was measured by bioluminescence imaging (BLI) by i.p. injection of 150 mg/kg D-luciferin or 150 ng Coelenterazine-h (Perkin Elmer, Waltham, MA) and imaged using the IVIS imaging system (Perkin Elmer). Photon emission was analyzed by whole-body region of interest (ROI), and the signal was measured as average radiance (photons/second/cm^2^/steradian).

### Western blot analysis

Non-transduced and gene-modified T cells were harvested and lysed, and lysates were quantified for protein content. Protein lysates were electrophoresed on 10% sodium dodecyl sulfate–polyacrylamide gels and immunoblotted with primary antibodies to β-actin (1:1000, Thermo), caspase-9 (1:400, Thermo), and MyD88 (1:200, Santa Cruz). The secondary antibodies used were HRP-conjugated goat anti-rabbit or mouse IgG antibodies (1:500, Thermo). Membranes were developed using the SuperSignal West Femto Maximum Sensitivity Substrate Kit (Thermo, 34096) and imaged using a GelLogic 6000 Pro camera and CareStream MI software (v.5.3.1.16369).

### Analysis of in vitro and in vivo cytokine production

Cytokine production of IFN-γ, IL-2, and IL-6 by T cells modified with iMC or control vectors was analyzed by ELISA or cytometric bead array as recommended (eBioscience, San Diego, CA or Becton Dickinson, East Rutherford, NJ). In some experiments, cytokines were analyzed using a multiplex array system (Bio-Plex MAGPIX; Bio-Rad, Hercules, CA or Milli-Plex; Millipore, Burlington, MA).

### Statistics

Data are represented as mean ± SEM. Data were analyzed using Mann–Whitney statistical comparisons to determine significant differences between groups. One-way ANOVA followed by Bonferroni’s multiple comparison test was used to compare multiple treatment groups. Two-way ANOVA followed by Bonferroni’s test was used to assess the statistical significance of the differences in tumor growth between multiple treatment groups at different time points. Survival was recorded by Kaplan–Meier graphs, with significance determined by the log-rank test. Nonparametric assumptions were made for the data analyzed. Data were analyzed using GraphPad Prism v5.0 or newer software (GraphPad, La Jolla, CA).

## Results

### Inclusion of MyD88/CD40 endodomain within the CAR architecture provides costimulation but diminishes CAR activity in vivo

**Fig. 1:**
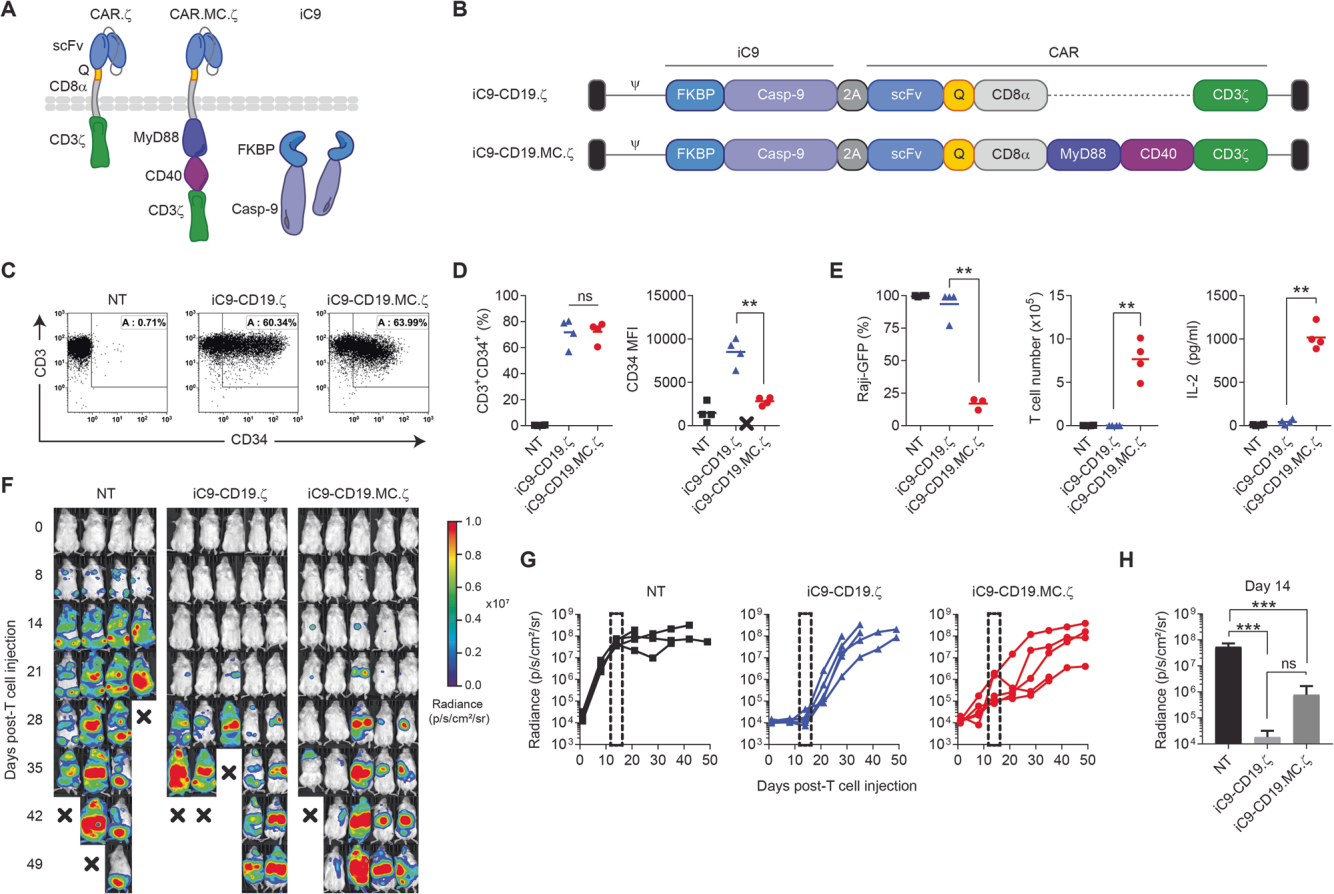
MC domains expressed in cis with CD3ζ provide costimulation but lower CAR activity. **a**, **b** Schematic comparing a conventional first-generation CAR with an enhanced CAR, including the signaling domains from MC, expressed in cis with the CD3ζ intracellular domain. These bicistronic vectors also express iC9 in the first position of the retroviral vector. **c**, **d** CD3^+^CD34^+^ expression using flow cytometry was used to measure the transduction efficiency and CAR mean fluorescence intensity (MFI). **e** Potency of non-transduced (NT) T cells or T cells modified with either iC9-CD19.ζ or iC9-CD19.MC.ζ were assessed in 7-day coculture assays with CD19^+^ Raji-EGPFluc tumor cells at a 1:1 effector-to-target (E:T) ratio. Tumor and T-cell frequency (%) were analyzed by flow cytometry, and IL-2 production was assessed by ELISA after 48 h of coculture. **f**, **g** Immune-deficient NSG mice were engrafted with CD19^+^ Raji-EGFPluc tumor cells on day 0 via tail vein injection and subsequently treated with NT, iC9-CD19.ζ, or iC9-CD19.MC.ζ-modified T cells on day 4 post-tumor injection. Mice were assessed by bioluminescence imaging (BLI) on an approximately weekly basis to determine tumor growth and CAR-T cell activity. **h** Analysis of tumor BLI was assessed on day 14 post-T-cell injection. ** represents *P*-value < 0.01; *** represents *P*-value < 0.005

To provide CAR-T cells with MC costimulation while retaining the ability to use the rimiducid-activated iC9 safety switch, we constructed a bicistronic retroviral vector encoding iC9 followed by a CD19-specific CAR comprising a truncated MyD88 (lacking the TIR domain) and CD40 (lacking the extracellular domain) upstream of the CD3ζ signaling element and compared it to a first-generation, iC9-expressing CD19 CAR (Fig. 1a, b). Transduction of primary T cells showed equivalent CAR transduction efficiencies for CD19.ζ and CD19.MC.ζ constructs (71 ± 10% vs. 72 ± 8%, respectively); however, CAR surface expression (MFI) was significantly diminished with the addition of MC (MFI 8513 ± 1587 vs. 2824 ± 455; *p* < 0.005) (Fig. 1c, d). Construction of additional vectors expressing MC, or only MyD88 or CD40 revealed that MyD88 lowered CAR expression levels, but not transduction efficiency, suggesting that expression of MyD88 within the CAR led to CAR instability at the membrane (Supplementary Figure 1). Despite reducing CAR cell surface levels, inclusion of the MC signaling domains still enhanced CAR activity against CD19-expressing Raji tumor cells by increasing CAR-T proliferation and IL-2 cytokine production over CD19.ζ-only modified T cells (23-fold increase; *p* < 0.0001) (Fig. 1e). We subsequently evaluated CD19-targeted CAR activity using NSG mice engrafted with CD19^+^ Raji tumors. Here, intravenous injection of 5 × 10^6^ iC9-CD19.ζ or iC9-CD19.MC.ζ-modified T cells showed significant tumor control over non-transduced (NT) T cells (***p ≤ 0.0001 at day 14) but did not produce durable responses (Fig. 1f, g). Importantly, the addition of MC did not improve antitumor activity compared with a first-generation construct (Fig. 1h). These data suggest that MyD88 is not compatible with normal expression as a costimulatory domain within the CAR architecture.

### Constitutive expression of MC outside the CAR-T molecule provides robust costimulation while preserving CAR expression

**Fig. 2:**
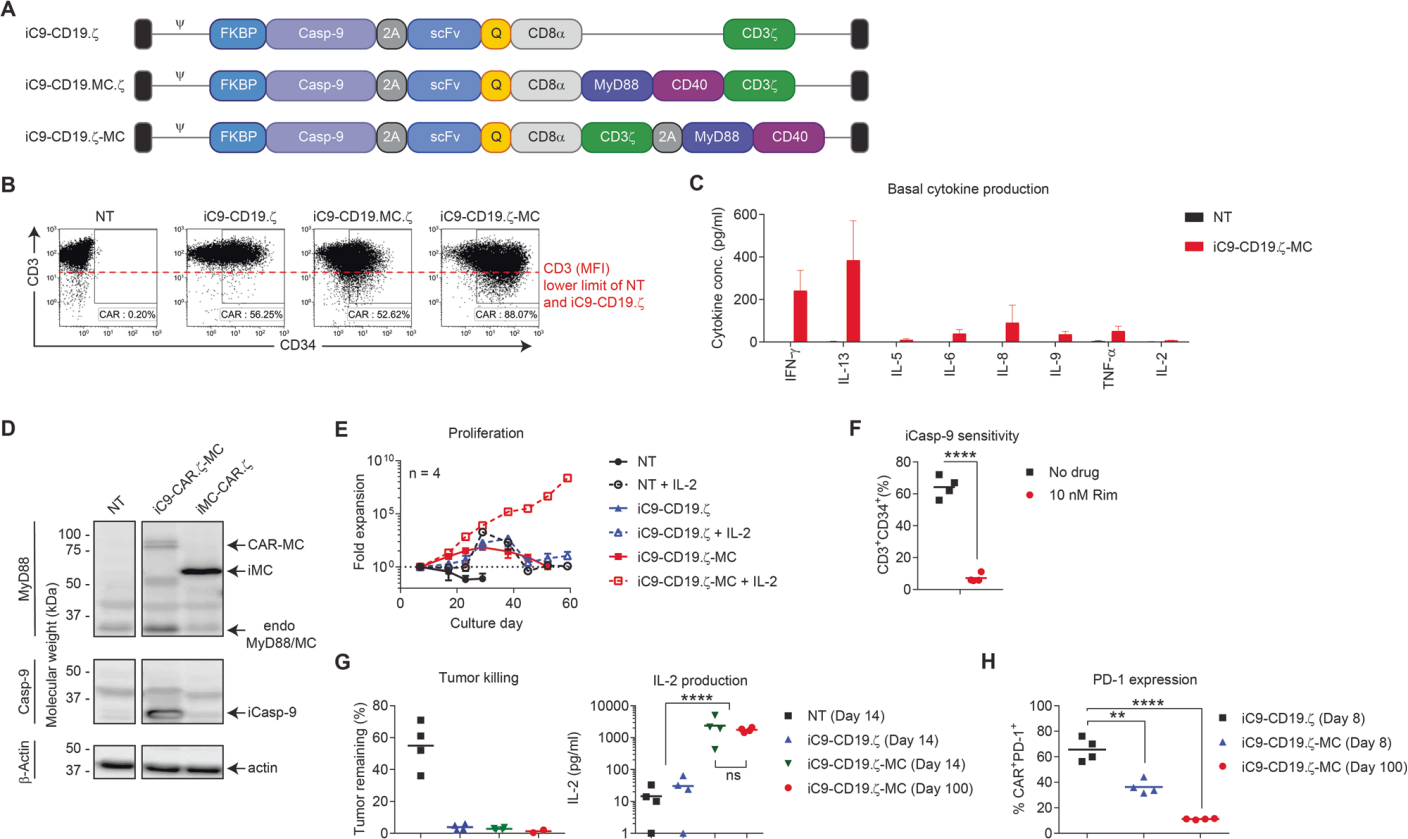
Constitutive expression and partial 2A cleavage results in CAR-T cells with high basal activity. **a** A tricistronic vector encoding iC9, CD19.ζ, and MC was generated with the transgenes separated by T2A and P2A ribosomal skipping sequences, respectively, and compared with iC9-CD19.ζ and the previously described iC9.CD19.MC.ζ vectors. **b** Non-transduced (NT) and T cells transduced with each vector were compared for transduction efficiency and CAR MFI. The dotted red line labeled “CD3 (MFI)” indicates the approximate lower limit of CD3 expression on NT and iC9-CD19.ζ T cells. **c** NT and iC9-CD19.ζ-MC-modified T cells were assessed for basal cytokine production after 48 h by a cytokine multiplex. **d** A western blot analysis was performed on NT, iMC-CD19.ζ, and iC9-CD19.ζ-MC using an anti-MyD88, anti-Casp-9, and β-actin antibodies demonstrating fusion of CAR-MC and high levels of iCasp-9 expression. **e** Long-term cultures were established to assess the contribution of basal activation to CAR-T survival and proliferation with or without exogenous cytokine support (100 U/ml IL-2), showing that CAR-MC basal activity is sufficient to drive T-cell expansion in the presence of IL-2. **f** Long-term cultured (100 days) iC9-CD19.ζ-MC-modified T cells remained sensitive to iC9-induced apoptosis when exposed to 10 nM rimiducid, **g** continued to show cytotoxicity against Raji tumor cells in 7-day coculture assays and produced IL-2, and **h**) downregulated PD-1 expression compared with iC9-CD19.ζ-modified T cells. ** and **** represent a *P*-value of <0.01 and <0.001, respectively

To determine if MC could still be useful as a constitutively expressed costimulatory module to drive T-cell proliferation, we expressed MC outside of the CAR molecule using a tricistronic gene expression approach using an additional 2A sequence (Fig. 2a). Removing MC from the CAR and expressing it as a separate polypeptide (iC9-CD19.ζ-MC) restored CAR expression levels on gene-modified T cells (Fig. 2b), while downregulating endogenous T-cell receptor (TCR) levels, consistent with T-cell activation (Fig. 2b). Indeed, iC9-CD19.ζ-MC-modified T cells secreted pro-inflammatory cytokines, including IFN-γ, IL-5, IL-6, IL-8, IL-9, and TNF-α in the absence of antigen stimulation, supporting that MC was providing a constitutive T-cell activating signal in this construct (Fig. 2c). Importantly, iC9-CD19.ζ-MC did not trigger IL-2 secretion in the absence of CAR-T engagement. By probing MyD88 expression using western blot analyses in NT, iC9-CD19.ζ-MC-modified and T cells transduced with an inducible MyD88/CD40 CD19-CAR vector (iMC-CD19.ζ), we were able to detect both a fast-migrating (~30 kDa) and a fainter slow-migrating (~90 kDa) fragment in iC9-CD19.ζ-MC-transduced T cells, suggesting that MC was incompletely separated from the membrane-associated CAR.ζ molecules expressed in this context, presumably due to inefficient 2A ribosomal skipping (Fig. 2d) [15]. To understand whether MC-mediated constitutive T-cell activation resulted in autonomous CAR-T proliferation, we cultured NT, iC9-CD19.ζ, or iC9-CD19.ζ-MC-modified T cells in the presence or absence of exogenous IL-2 (100 U/ml). In the presence of IL-2, this MC-CAR tethering could induce sustained, extensive (over 10^8^) expansion of CAR-T cells after 60 days of culture, yet iC9-CD19.ζ-MC-expressing CAR-T cells failed to survive in the absence of IL-2, reducing the risk of autonomous growth (Fig. 2e).

Despite prolonged culture, these MC-modified cells remained functional and sensitive to rimiducid. Long-term cultured (100 days) iC9-CD19.ζ-MC-transduced T cells remained highly sensitive to iC9-induced apoptosis when exposed to rimiducid (Fig. 2f), retained cytotoxic activity, and secreted high-level IL-2 in coculture assays with CD19^+^ target cells similar to T cells cultured for a shorter period (14 days) (Fig. 2g). Interestingly, iC9-CD19.ζ-MC-modified T cells showed a decrease in PD-1 expression compared with a first-generation CAR, suggesting that constitutive MC activity may reduce the sensitivity of iC9-CD19.ζ-MC T cells to PD-L1 expression in the tumor microenvironment (Fig. 2h). Additionally, long-term culture of iC9-CD19.ζ-MC-modified T cells shows that these cells exhibited a similar T-cell subset distribution to that of first-generation CD19-CAR-T cells (CD45RA^+^CD62L^+^ T_N_, CD45RA^–^CD62L^+^ T_CM_, CD45RA^–^CD62L^–^ T_EM_, and CD45RA^+^CD62L^–^ T_EMRA_). However, after 100 days in culture, T_EM_ (CD3^+^CD45^–^CD62L^–^) cells were the predominant subtype present in iC9-CD19.ζ-MC-modified T-cell cultures (Supplementary Figure 2). Thus, iC9-CD19.ζ-MC is a constitutively active CAR construct with sustained proliferative capacity in the presence of antigen stimulation or exogenous IL-2 but is responsive to controlled elimination through the iC9 safety switch.

### Constitutive MC-CAR-T demonstrates robust antitumor activity against CD19^+^ lymphomas in animals

**Fig. 3:**
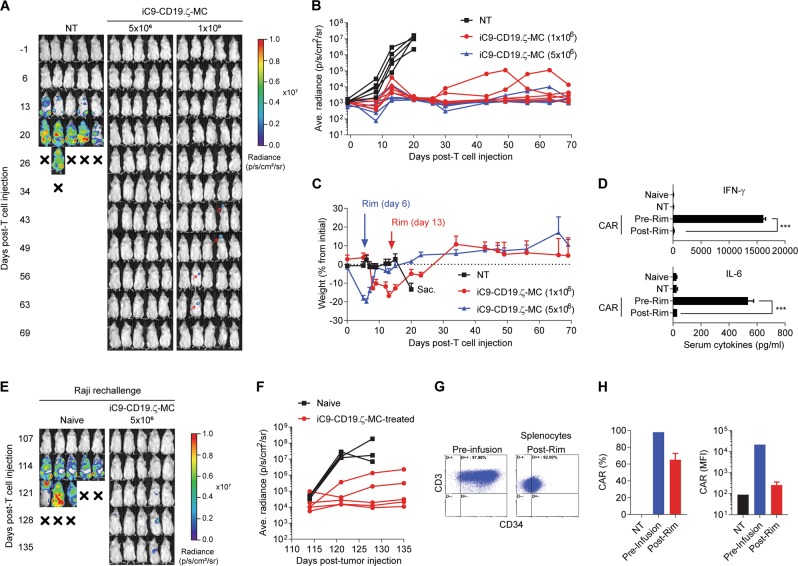
MC-dependent basal expression results in potent antitumor activity and the associated toxicity. **a**, **b** NSG mice engrafted with CD19^+^ Raji-EGFPluc tumor cells were treated with 5 × 10^6^ non-transduced (NT) or 1 × 10^6^ or 5 × 10^6^ iC9-CD19.ζ-MC-modified T cells via i.v. injection after 7 days. Tumor growth was assessed by bioluminescence imaging (BLI) on a weekly basis for 70 days post-tumor challenge. **c** Weight of control (NT) and CAR-T-treated animals was measured to assess CAR-related toxicities. Mice exhibited a >20% reduction in weight on days 6 and 13 after receiving 5 × 10^6^ and 1 × 10^6^ iC9-CD19.ζ-MC-modified T cells, respectively. At this time, a single injection of 5 mg/kg rimiducid was administered i.p., which promptly resolved the toxicity. **d** Serum cytokine levels were assessed in naive (untreated), NT, and CAR-treated animals before and 24 h after rimiducid injection, showing high levels of hIFN-γ and hIL-6 prior to drug administration and returning to background levels following the activation of the iC9 safety switch. **e**, **f** Naive mice and mice that received CAR-T cells and rimiducid were subsequently rechallenged with Raji-EGFPluc tumor cells, demonstrating that residual iC9-CD19.ζ-MC-modified T cells can effectively control tumor outgrowth. **g**, **h** Twenty-five days post-tumor rechallenge, mice were euthanized and the splenocytes were analyzed for the presence of CAR-T cells (CD3^+^CD34^+^) by flow cytometry and compared with the original product for frequency and CAR expression (mean fluorescence intensity, MFI). *** represents a *P*-value < 0.005

CD19-targeted CAR-T cells expressing constitutive MC were evaluated for efficacy in vivo using immune-deficient NSG mice engrafted with the CD19^+^ Raji cell line, modified with the EGFPluc transgene (Raji-EGFPluc) to allow in vivo bioluminescence imaging (BLI). Raji tumor cells grew rapidly in mice treated with 5 × 10^6^ NT T cells, requiring euthanasia by day 21 due to hind-leg paralysis (Fig. 3a). In contrast, mice treated with 1 × 10^6^ or 5 × 10^6^ iC9-CD19.ζ-MC-modified T cells showed early tumor control, which corresponded to acute weight loss in a CAR-T cell dose-dependent manner (Fig. 3a–c). However, CAR-related toxicity was successfully resolved by the administration of 5 mg/kg rimiducid (i.p.) when the mice reached >10% loss in body weight (from initial measurement) (Fig. 3c). Serum samples taken before and after rimiducid treatment showed high pre-rimiducid levels of human cytokines, including IFN-γ and IL-6, which reverted to baseline levels by 24 h post-rimiducid exposure (Fig. 3d). Importantly, long-term tumor control was not compromised by the activation of the iC9 safety switch, where all the CAR-T-treated mice remained tumor-free (by BLI) out to 70 days (Fig. 3a, b). As observed in a previous study based on iC9 to lower CAR-T activity [16], animals were resistant to subsequent tumor challenge compared with naive mice, presumably due to residual T cells expressing reduced levels of iC9-CD19.ζ-MC (Fig. 3e, f). Consistent with this hypothesis, residual CAR-T cells could be detected in the spleens of rimiducid-treated animals (Fig. 3g, h). Furthermore, a comparison versus first- (iC9-CD19.ζ) and second-generation (iC9-CD19.28.ζ and iC9-CD19.BB.ζ) CAR constructs showed that antitumor activity was not impaired compared with these alternative CD19 CARs in this animal model, despite the need to deploy iC9 with rimiducid to control the toxicity in animals treated with iC9-CD19.ζ-MC-modified T cells (Supplementary Figure 3).

**Fig. 4:**
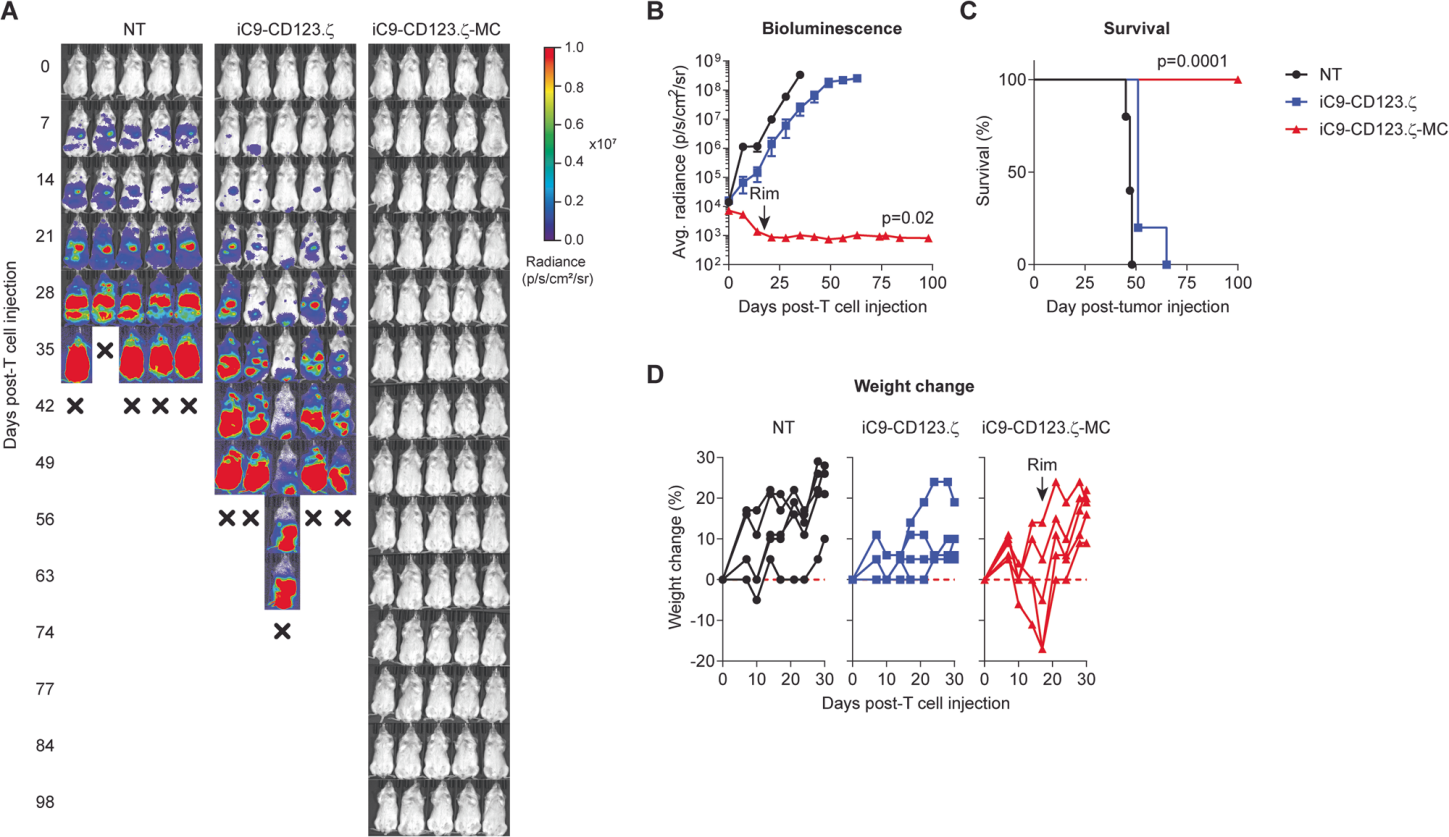
iC9-CD123.ζ-MC-modified T cells effectively eliminate CD123^+^ leukemia while managing toxicity with iC9. **a**, **b** NSG mice were engrafted with CD123^+^ THP-1-EGFPluc tumor cells and subsequently treated with 2.5 × 10^6^ non-transduced (NT) or iC9-CD123.ζ-MC-modified T cells. **b** Tumor growth was evaluated on a weekly basis using BLI measurements and **c** 100-day survival was assessed showing a robust and long-term antitumor activity from T cells expressing constitutively active MC compared with iC9-CD19.ζ-modified T cells. **d** Like CD19-targeted, MC-enhanced CARs, iC9-CD123.ζ-MC-expressing T cells showed similar toxicity in NSG animals, which could be resolved by administration of rimiducid without affecting antitumor activity

To evaluate the versatility of this approach, we subsequently performed in vivo evaluation of the constitutive MC CAR-T platform targeting a CD123^+^ myeloid cell line (THP-1-EGFPluc) and compared it with NT and T cells modified with an iC9-enabled, first-generation CAR (iC9-CD123.ζ) (Fig. 4a). THP-1-EGFPluc showed rapid outgrowth in mice treated with control T cells, resulting in termination by day 35, while iC9-CD123.ζ-modified T cells showed modest antitumor activity, delaying tumor growth by ~2 weeks (Fig. 4a, b). However, addition of MC to the construct (iC9-CD123.ζ-MC) provided durable antitumor responses (> day 100 post-T-cell injection) (Fig. 4a–c). As observed with iC9-CD19.ζ-MC-expressing T cells, 3/5 (60%) of the mice experienced acute toxicity in the form of cachexia by day 14 post-T-cell treatment, which could be resolved by rimiducid administration without affecting tumor control (Fig. 4d). Thus, in multiple tumor models, constitutively active MC-driven CAR-T cells demonstrate robust antitumor effects, but cause cachexia in mice due to their high basal activity, necessitating iC9-mediated toxicity mitigation.

### Rimiducid titration allows partial ablation of constitutive CAR-T activity and modulates systemic cytokine levels

**Fig. 5:**
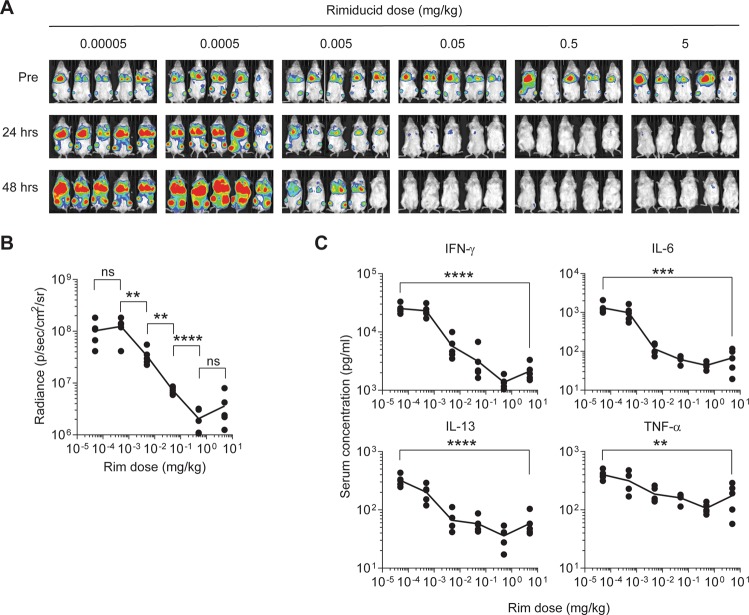
Frequency of iC9-CD19.ζ-MC-modified T cells and cytokine production can be modulated by titration of rimiducid. **a** NSG mice were engrafted with non-modified CD19^+^ Raji tumor cells and subsequently treated with 5 × 10^6^ T cells transduced with iC9-CD19.ζ-MC and EGFPluc retroviral vectors on day 7 post-tumor injection. CAR-T cell levels were assessed by BLI before and 24 and 48 h after i.p. injection of rimiducid (0.00005, 0.0005, 0.005, 0.05, 0.5, and 5 mg/kg). **b** CAR-T cell BLI and **c** serum cytokine levels of IFN-γ, IL-6, IL-13, and TNF-a at 24 h post-rimiducid treatment were measured. **, ***, and **** represent a *P*-value of <0.01, 0.005, and 0.001, respectively

iC9-CD19.ζ-MC-modified T cells show a high basal activation state, which is linked to their antitumor activity. While administration of high-dose rimiducid (5 mg/kg) allowed persistence of low-level CAR-T cells, we considered that rimiducid titration could permit retention of more gene-modified T cells while mitigating cytokine-related toxicities. To test this, we co-transduced T cells with iC9-CD19.ζ-MC and EGFPluc and administered them into Raji-bearing mice. Following the onset of cachexia (>10% body weight loss), a log titration of rimiducid (5–5 × 10^–5^ mg/kg) was administered as a single i.p. injection (Fig. 5a). As previously observed [16], CAR-T cell BLI was reduced in a rimiducid dose-dependent manner (Fig. 5b). CAR-T cell reduction corresponded to decreased serum cytokine levels (i.e., IL-6, IFN-γ, and TNF-α) (Fig. 5c). With this highly active construct, rimiducid titration could be selectively modulated to minimize excessive activity while maximizing therapeutic potency.

### MC basal activity is required for CAR-T cell expansion in vivo

**Fig. 6:**
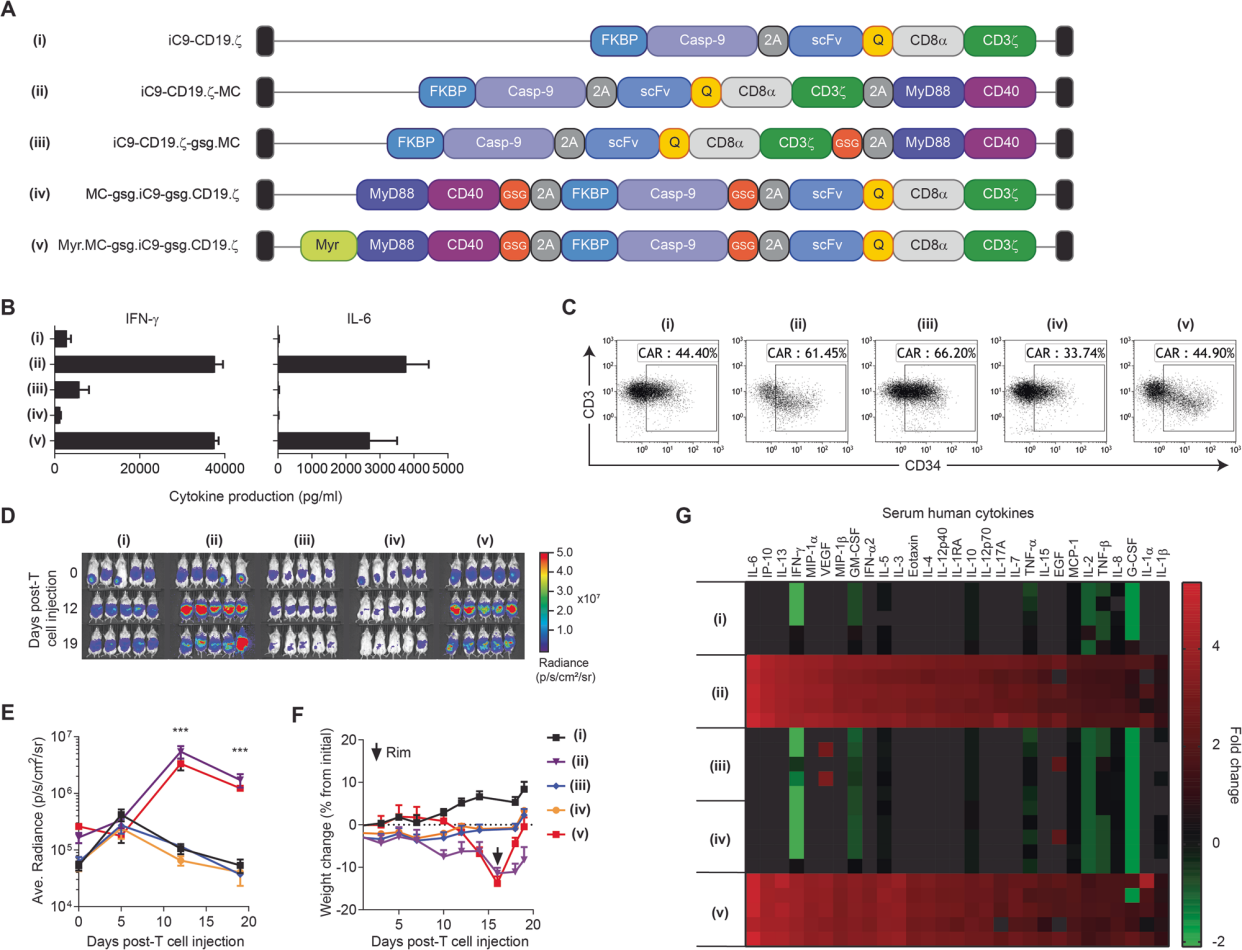
CAR-MC basal activity required for T-cell expansion and cytokine-related toxicity. **a** Additional vectors were designed to better understand the contribution of CAR-MC basal effects on antitumor activity and cytokine-related toxicities in animal models. iC9-CD19.ζ (i) and iC9-CD19.ζ-MC (ii) were compared with constructs bearing high-efficiency 2A cleavage peptides (GSG-2A) (iii) or with MC moved to the first position to eliminate CAR-MC fusion pairing (iv). In addition, a vector was constructed with a myristoylated MC domain to enhance basal activity by tethering the signaling domain to the cell membrane (v). **b** Basal activity of CAR-modified T cells was assessed by measuring IFN-γ and IL-6 in the absence of an antigen. To measure CAR-T cell expansion, **c** T cells were co-transduced with a CAR vector and ONL and subsequently administered to CD19^+^ Raji-bearing mice, **d**, **e** where CAR-T cell expansion was measured on days 0 (post-T-cell injection), 12, and 19. **f** Toxicity from MC-based CAR-T cells was assessed by measuring weight loss. Groups exhibiting > 10% weight loss were treated with a single injection of rimiducid at 0.5 mg/kg. **g** Serum levels of cytokines and chemokines were assessed on day 7 post-CAR-T cell injection. Changes in cytokine/chemokine levels are represented as fold change from pre-CAR-T cell infusion samples

As shown in Fig. 2d, inefficient 2A cleavage appeared to result in MC association with some CAR molecules. This observation led us to hypothesize that the small fraction of membrane-proximal MC fused to CAR.ζ accounts for the basal signaling activity seen, subsequently driving CAR-T cell proliferation and antitumor activity. To test this hypothesis, we generated additional constructs incorporating GSG-linked 2A sequences (GSG linker) [15, 17] to more efficiently separate MC from the CAR, as well as evaluating the relocation of MC to the first gene position to exclude intracellular attachment to the CD3ζ chain (Fig. 6a). In addition, we attempted to replicate high basal signaling that results from the juxtaposition of MC to the membrane by including a myristoylation-targeting domain to increase the inner membrane association of MC [18]. We subsequently measured basal cytokine production from transduced T cells. Cytokine analysis showed that improved GSG-linked 2A cleavage and moving MC to the 5′ position dramatically reduced basal IFN-γ and IL-6 production, while partial CAR attachment (iC9-CD19.ζ-MC) and membrane-associated MC (Myr-MC) revealed high levels of cytokine secretion, supporting the hypothesis (Fig. 6b). Interestingly, when using CAR-T cells co-modified with ONL to measure T-cell levels in vivo, high tonic signaling was associated with rapid expansion at days 12 (~fourfold; *p* < 0.005) and 19 (~eightfold; *p* < 0.001) post-CAR-T cell injection (Fig. 6c–e).

While high basal activity enhanced CAR-T expansion, it was also associated with cachexia, which required rimiducid infusion to activate iC9 (Fig. 6f). The profile of CAR-T-produced human cytokines in these animals showed that iC9-CD19.ζ-MC and MyrMC-iC9-CD19.ζ-modified T cells produced high levels of a diverse number of pro-inflammatory cytokines compared with constructs with low basal CAR-T activity (Fig. 6g). In addition, a comparison with an inducible MC system (i.e., iMC^5,6^), using the CD19^+^ Raji tumor model indicates that high basal activity is necessary for prolonged antitumor efficacy, but is also associated with cachexia in this model (Supplementary Figure 4). Together, these data suggest that basal activation by MC can enhance CAR-T activity and proliferation in vivo, but that cytokine production from rapidly proliferating T cells can cause undesired side effects necessitating the use of rimiducid and iC9.

### CD8 selection of iC9-CD19.ζ-MC-modified T cells abrogates toxicity by reducing cytokine production

To better understand the cause of cachexia following i.v. administration of iC9-CAR.ζ-MC-modified T cells, we performed additional experiments against CD19^+^ Daudi tumors and attempted to counteract weight loss using neutralizing antibodies to three overexpressed inflammation-associated cytokines, human IL-6, IFN-γ, and TNF-α, all of which can cross-react with murine cytokine receptors. Here, tumor-bearing mice were treated with 5 × 10^6^ iC9-CD19.ζ-MC-transduced T cells, and following >10% weight loss, intervention with either a single i.p. dose of rimiducid (0.5 mg/kg) or vehicle, or twice weekly injections of 100 μg per mouse anti-hIFN-γ, hIL-6, or hTNF-α was initiated (Supplementary Figure 5A). Interestingly, only anti-hTNF-α treatment was able to protect mice from further health decline to the same level of protection as activating the iC9 safety switch (Supplementary Figure 5B). The protection by anti-hTNF-α treatment from further weight decline was associated with only a modest, nonsignificant reduction in serum hTNF-α levels consistent with blockade of ligand–receptor interactions, rather than mediating the clearance of antibody-bound hTNF-α (Supplementary Figure 5C). In contrast, activation of iC9 with rimiducid significantly reduced serum concentrations of hTNF-α. Analogous to the use of iC9, control of the toxicity with anti-hTNF-α did not affect antitumor activity of the CAR-T cell therapy (Supplementary Figure 5A). Thus, cytokine blockade provides a second effective mechanism to resolve the toxicity of this potent approach.

**Fig. 7:**
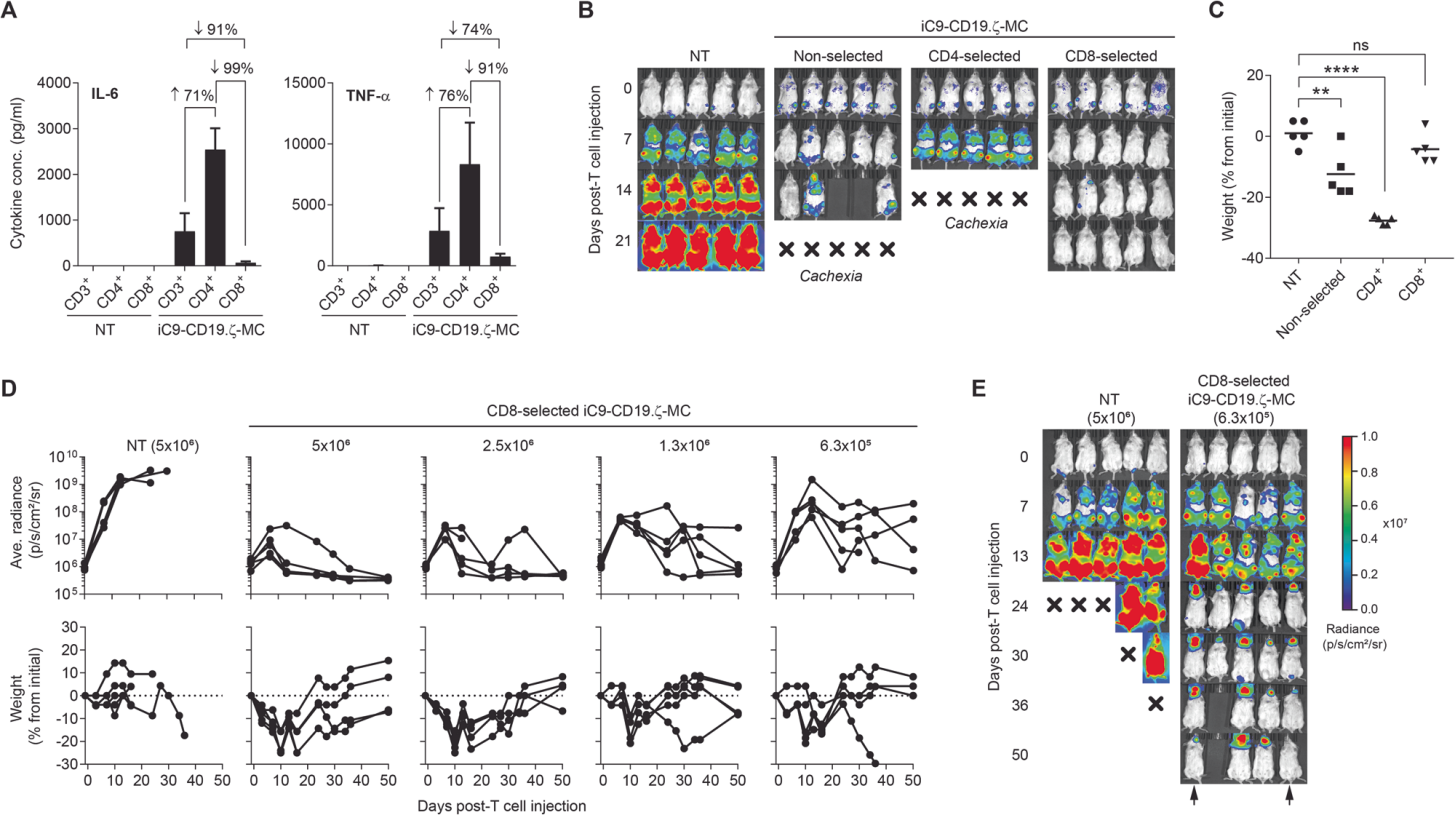
Enrichment of CD8^+^ T cells reduces cytokine-related toxicities and amplifies the antitumor effect of iC9-CD19.ζ-MC-modified T cells. **a** Non-transduced (NT), unselected (CD3^+^), CD4, and CD8-selected iC9-CD19.ζ-MC-modified T cells were cultured with CD19^+^ Raji tumor cells and measured for IL-6 and TNF-α secretion after 48 h. **b**, **c** NT, nonselected, CD4, and CD8-selected CAR-T cells were infused into CD19^+^ Raji-EGFPluc-bearing NSG mice and tumor growth was measured by bioluminescence imaging (BLI). Mice that exhibited severe toxicity post-CAR-T cell infusion were euthanized. Rimiducid to activate iC9 was not administered to any of the animals. **d** Mice bearing CD19^+^ Raji-EGFPluc tumors were treated with 6.3 × 10^5^, 1.3 × 10^6^, 2.5 × 10^6^, or 5 × 10^6^ CD8-selected iC9-CD19.ζ-MC-modified T cells on day 4 and measured for BLI and weight loss. None of the groups received rimiducid to control CAR-related toxicity. **e** Representative bioluminescence images of mice receiving 5 × 10^6^ CD8-selected iC9-CD19.ζ-MC-modified T cells. Arrows denote late resolution of intracranial tumors. ** and **** represent a *P*-value of <0.01 and 0.001, respectively

As T-cell subsets can have different properties, we speculated that subset purification might provide a third avenue for controlling the toxicity. It is well-established that CD4^+^ T cells produce high levels of pro-inflammatory cytokines following antigen recognition. Because TNF-α and possibly other cytokines contributed to cachexia following i.v. injection of iC9-CD19.ζ-MC-modified T cells, we sought to determine if the selection of CD8^+^ T cells (or depletion of CD4^+^ cells) could lessen the toxicity while preserving antitumor activity. Here, NT and CAR-modified T cells were purified into CD4^+^ and CD8^+^ T cells using magnetic bead selection (Supplementary Figure 6A). Nonselected and selected T cells were tested for purity and transduction efficiency. Whereas nonselected CAR-T cells had a CD4:CD8 ratio of 1:2, following selection, they were 99% and 90% pure for CD4 and CD8-selected T cells, respectively (Supplementary Figure 6C). iC9-CD19.ζ-MC transduction was equivalent in both selected and nonselected gene-modified T cells (~62% CD3^+^CD34^+^) (Supplementary Figure 6B). We subsequently performed coculture assays against Raji tumor cells and measured IL-6 and TNF-α production at 48 h and found that CD4-selected CAR-T cells produced 71% and 76% higher levels of IL-6 and TNF-α compared with unselected CAR-T cells, whereas CD8-selected CAR-T cells produced 99% and 91% less of these molecules, respectively (Fig. 7a). To test whether this modification could reduce cachexia, we subsequently infused NT, nonselected, CD4 or CD8-enriched iC9-CD19.ζ-MC-modified T cells into Raji-EGFPluc-bearing NSG mice and observed that although nonselected and CD4-enriched iC9-CD19.ζ-MC CAR-T cells showed improved tumor control over NT T cells (Fig. 7b), these mice rapidly developed cachexia by day 7 post-CAR-T cell injection (Fig. 7c). In contrast, CD8-selected CAR-T cells demonstrated superior tumor control with minimal concomitant weight loss (Fig. 7b, c). Finally, we then performed a dose titration with CD8-enriched iC9-CD19.ζ-MC-modified T cells using the same animal model. Here, high cell doses (>2.5 × 10^6^ cells) rapidly controlled tumor outgrowth (Fig. 7d). While these animals did show some evidence of cachexia, iC9 activation with rimiducid was not required and all animals recovered ~2–3 weeks post-CAR-T cell injection (Fig. 7d). Treatment with lower doses of CD8-enriched CAR-T cells also showed tumor control, albeit with slower tumor-elimination kinetics (Fig. 7d). Importantly, as few as 6.3 × 10^5^ CD8^+^ cells controlled high-level tumor burden with a durable efficacy (Fig. 7e). These experiments suggest that CD8-enriched iC9-CD19.ζ-MC-modified T cells have potent antitumor efficacy with reduced cytokine-associated toxicity and may be helper T-cell independent.

## Discussion

This study describes an empirically discovered CAR architecture that utilizes high basal CAR signaling and costimulation (i.e., “always on”) to drive T-cell proliferation and potent antitumor activity against aggressive CD19^+^ and CD123^+^ lymphoma and leukemia cell lines. While CAR-T cells expressing constitutively active MC produced high cytokine levels that could cause toxicity (i.e., IFN-γ, TNF-α, and IL-6), activating iC9 with rimiducid quickly and effectively resolved this toxicity in animal models. Importantly, rimiducid could be titrated to “partially” eliminate iC9-expressing CAR-T cells, preserving long-term antitumor efficacy. In addition, using neutralizing antibodies, we elucidated that CAR-T cell-secreted cytokines were responsible for the observed cachexia. This finding led to the selection and infusion of enriched CD8^+^ effector T cells with lower cytokine secretion, which resulted in lower toxicity with increased antitumor effects in a CD4^+^ helper-independent manner.

Initially, we attempted to express MC in cis with CD3ζ, analogous to conventional CAR designs that rely on well-characterized costimulatory domains, such as CD28 and 4-1BB. However, MyD88 appeared to destabilize the CAR, lowering surface expression and decreasing in vivo antitumor activity (Fig. 1). Therefore, we subsequently attempted to express MC as a freestanding protein to provide continuous costimulation to CD19-specific CAR-T cells. This resulted in the restoration of CAR surface expression on modified T cells and improved antitumor activity (Fig. 2). Western blot analyses revealed additional sub-molar MC species indicative of the formation of fusion proteins, potentially caused by inefficient 2A-mediated peptide bond skipping between CAR.ζ and the MC molecule. We hypothesize that ligation of MC to a fraction of membrane-localized CAR molecules induces a signaling cascade that is responsible for basal activity but leaves enough unmodified CAR present to maintain CAR surface levels and potency. Indeed, the addition of a small GSG linker to the 5′ end of the 2A peptide to increase transgene protein separation curtails basal cytokine secretion and abolishes in vivo CAR-T cell proliferation (Fig. 6). Tethered to CD3ζ, MyD88/CD40 may act as a scaffold to recruit other signaling proteins (e.g., interleukin-1 receptor-associated kinase (IRAK) family) as a MyDDosome complex to induce basal signaling [19–22]. Alternatively, tonic signaling from the scFv, amplified by MyD88/CD40, could result in constitutive stimulation [23]. However, since the scFv tested in most of our studies, FMC63, is not associated with high tonic signaling, simple membrane localization of MC is the more likely explanation consistent with the comparable activity triggered by MC myristoylation (Fig. 6a, b).

Unlike previous reports of the deleterious effects of constitutive CAR signaling, MC costimulation did not appear to induce CAR-T cell exhaustion [23, 24]. Indeed, MC-enabled CAR-T cells could proliferate for more than 3 months without loss of cytotoxic function, IL-2 production, and importantly, responsiveness to iC9-mediated apoptosis (Fig. 2e–g). Long et al. showed that some CAR costimulatory domains, such as 4-1BB, were protective against cellular exhaustion derived from tonic signaling [23]. Others have shown, however, that 4-1BB can contribute to FAS-dependent cell death under tonic CAR conditions [25]. In contrast, MC appears to phosphorylate a broad and unique set of signaling pathways [5]. In addition to signaling through NF-κB [5, 6], MC activates Akt, which has been shown to enhance survival and proliferation of CAR-T cells [26]. Additional signaling nodes (e.g., AP-1, MAPK, and IRF) may also contribute to enhanced function. Our (Supplementary Fig. 3 and [5]) and other’s observations [6] suggest that MyD88/CD40-derived costimulation may be a more potent driver of CAR-T cell activity than CD28 or 4-1BB. Whether MyD88/CD40 overcomes the limitations of conventional costimulatory molecules in T cells expressing constitutively active CARs will be a topic of further investigation.

Highly active T-cell therapies are a risk for cytokine-related toxicities, which can be amplified further in patients with high tumor burden [27]. In this study, constitutive MC signaling in CAR-T cells resulted in acute cachexia following infusion, which was neither specific to the CAR target (i.e., CD19 or CD123) nor in the longer time frame typically seen with xenogeneic graft-versus-host disease. However, toxicity could be mitigated by activation of iC9 following a single rimiducid injection. As previously demonstrated, rimiducid titration resulted in partial elimination of MC-enabled CAR-T cells, without loss of antitumor activity [16]. Use of neutralizing blocking antibodies revealed that blockade of TNF-α decreased CAR-T cell-related toxicity, suggesting that depletion of cell subsets that produce high levels of pro-inflammatory cytokines (i.e., CD4^+^ T helper cells) could improve the therapeutic window for using a constitutive, MC-enabled CAR-T cell therapy. Indeed, purification of CD8^+^ T cells resulted in improved efficacy with minimal cytokine-related toxicity that did not require the use of rimiducid to mitigate toxicity. Interestingly, MC appeared to support the expansion of CAR-T cells in a CD4^+^ helper-independent manner, suggesting that in clinical application, purification of CD8^+^ T cells might decrease CRS independent of the inclusion of putative regulatory CAR-T cells [28]. Since the animal models used in this study lacked human-derived myeloid cells, further investigation of iC9-CD19.ζ-MC CAR-T cells using recently described preclinical models of CRS would yield additional translational insight [29, 30].

In summary, constitutive MC costimulation provides CARs targeting CD19 or CD123 with long-term proliferative potential and high antitumor efficacy in animal models of lymphoma and myeloid leukemias, respectively. MC-enabled CAR-T cells exhibit substantial basal activity and are associated with cytokine-related toxicities in immune-deficient mice, but this can be managed by deployment of the iC9 safety switch with rimiducid, selective blockade of inflammatory cytokines (TNF-α), or by selecting T-cell subsets (CD8^+^) with the propensity for lower cytokine secretion. Overall, we identified a novel, highly efficacious CAR-T platform.

## Supplementary information

Supplementary Figures and Legends
