# Supplementary material for: Constitutively active MyD88/CD40 costimulation enhances expansion and efficacy of chimeric antigen receptor T cells targeting hematological malignancies
Source: Leukemia. 2019 Feb 28;33(9):2195–207. doi: 10.1038/s41375-019-0417-9 (PMC6756044; doi:10.1038/s41375-019-0417-9)
Supplement: Supplementary file 1 — Supplementary Figures and Legends [file 41375_2019_417_MOESM1_ESM.pdf]

Supplementary Figure 1

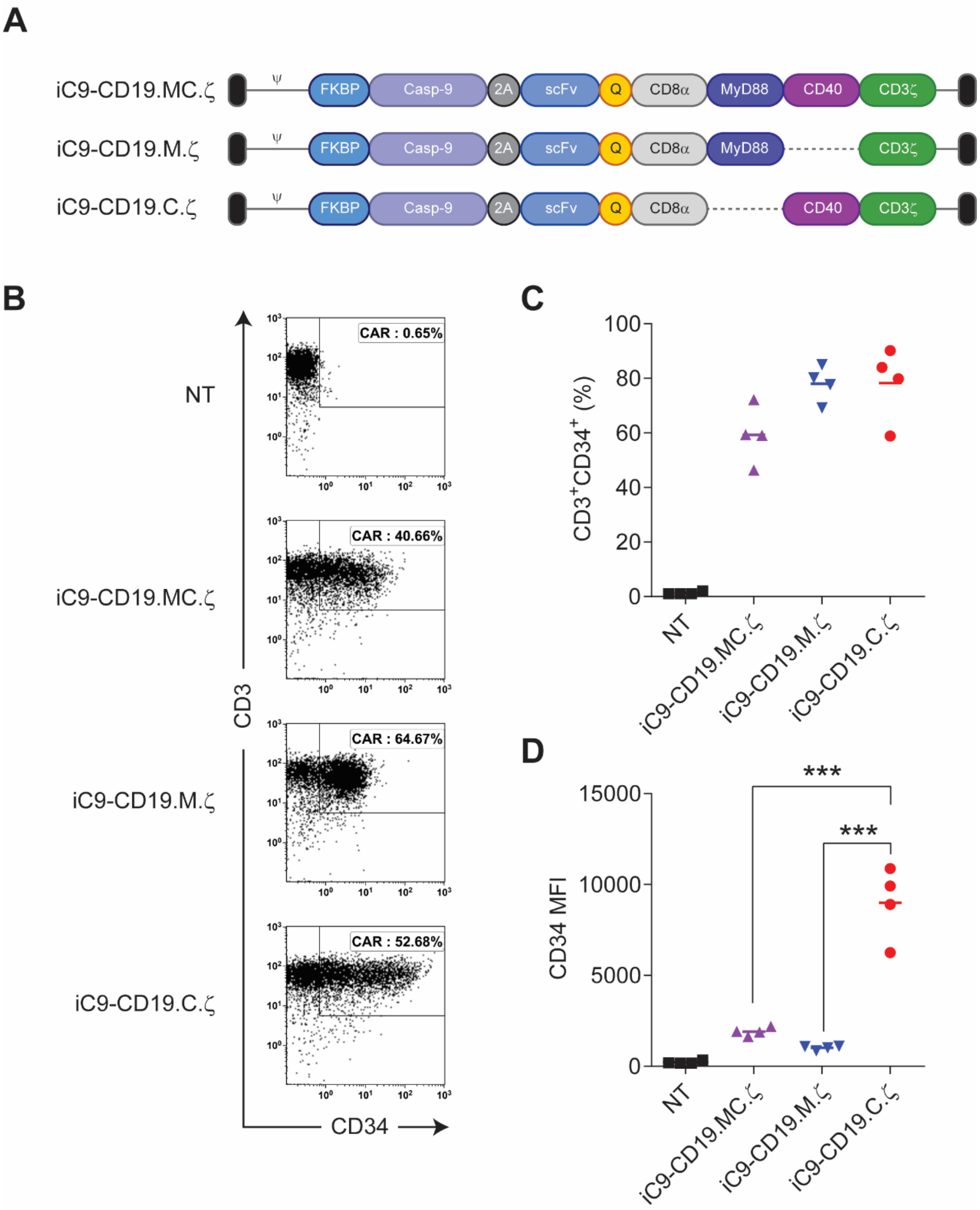

Supplementary Figure 1. *In cis* MyD88 architecture lowers CAR expression. A) To assess the impact of MC expression in CAR molecules, vectors were designed to encode MC, MyD88 (M) or CD40 (C) together with the cytoplasmic CD3ζ signaling domain. **B)** CAR expression was assessed by flow cytometry demonstrating that while transduction efficiency was unaffected, CAR levels were diminished by the inclusion of the MyD88 signaling domain. *** represents a P-value of <0.005.

Suppementary Figure 2

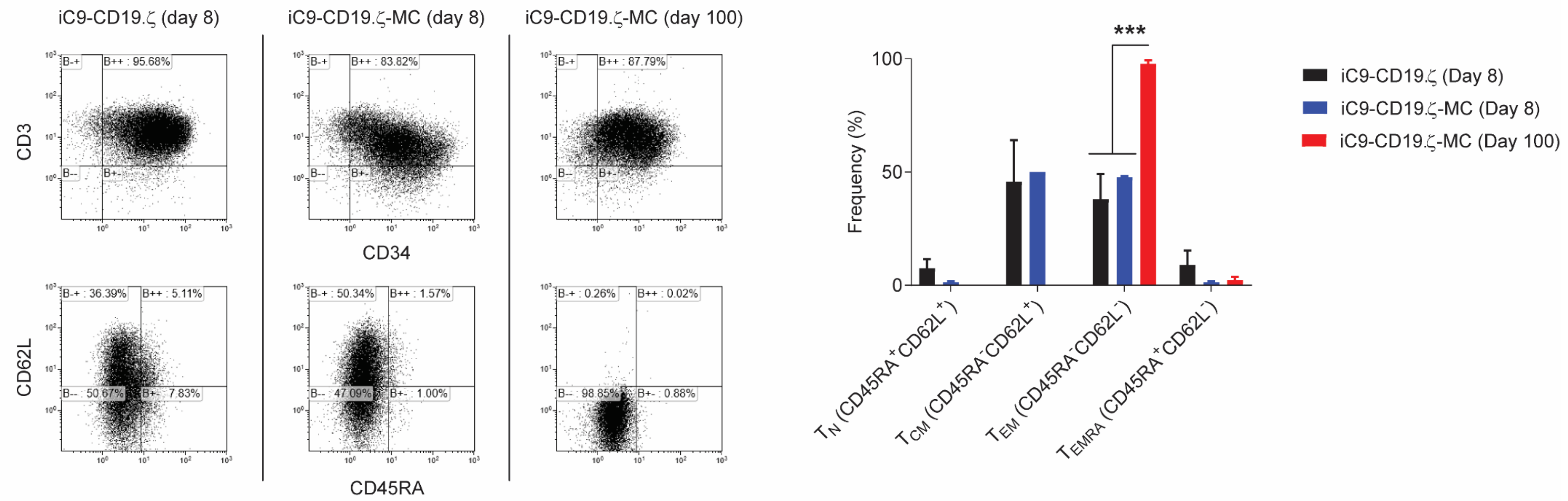

Supplementary Figure 2. Memory phenotype of modified T cells. Flow cytometric analysis of the memory phenotype using CD62L and CD45RA antibody staining for iC9-CD19.ζ and iC9-CD19.ζ-MC-modified T cells at day 8 and day 100 (iC9-CD19.ζ-MC) culture cells.

Supplementary Figure 3

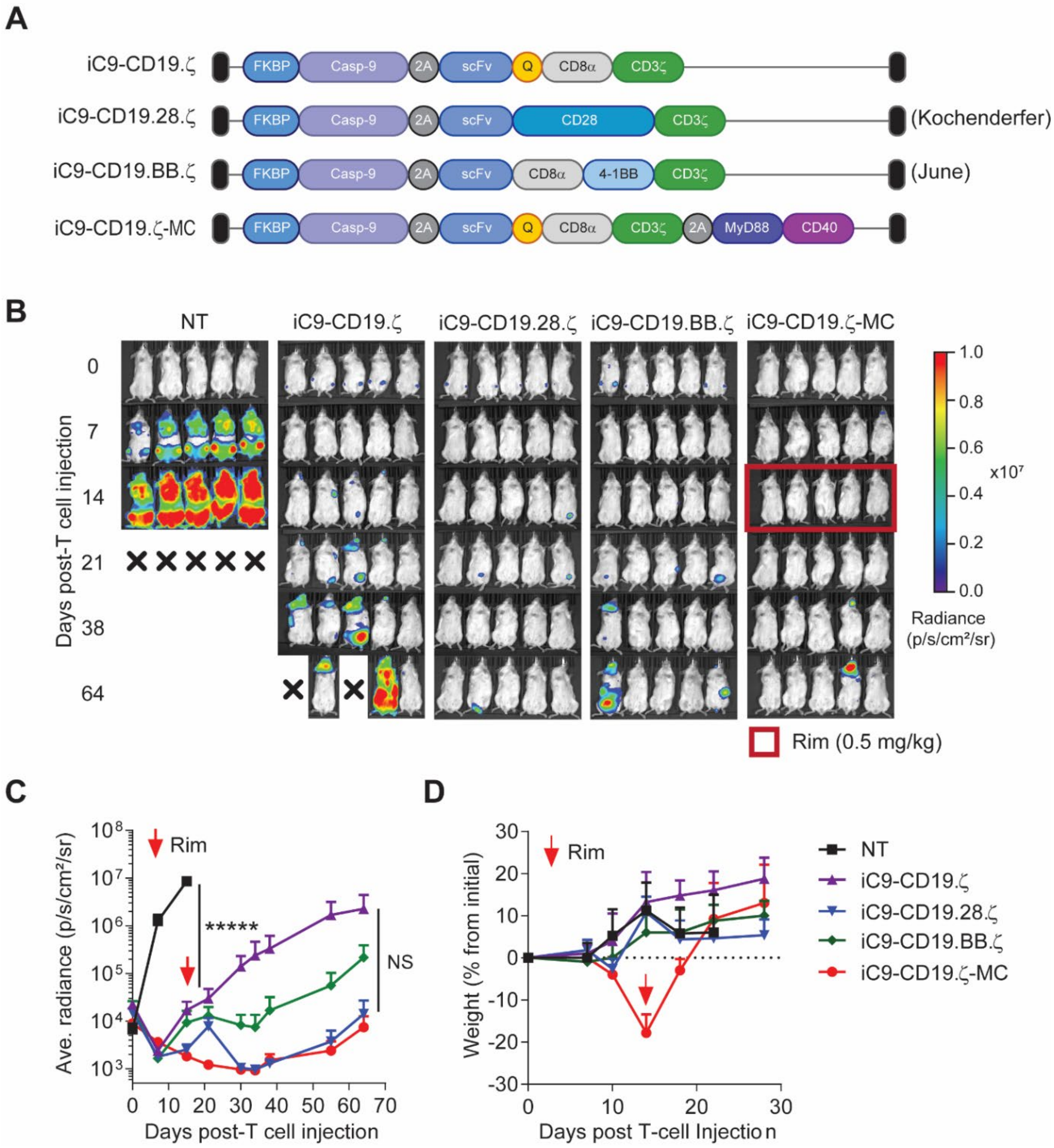

Supplementary Figure 3. MC-enhanced CAR-T cells show robust anti-tumor activity compared to CAR-T cells bearing CD28 and 4-1BB endodomains. A) Additional CD19-specific CAR constructs containing iC9 were developed using the CD28 and 4-1BB endodomains. Mice were engrafted with $CD19^{+}$ Raji-EGFPluc tumor cells and subsequently treated with non-transduced (NT) or CAR-modified T cells 7 days post-tumor engraftment. **B and C)** Tumor growth was measured by bioluminescent imaging on a weekly basis. **D)** Mice treated with iC9-CD19.ζ-MC-modified T cells were treated with 0.5 mg/kg rimiducid on day 12 (red arrow) to resolve acute CAR-related weight loss.

Supplementary Figure 4

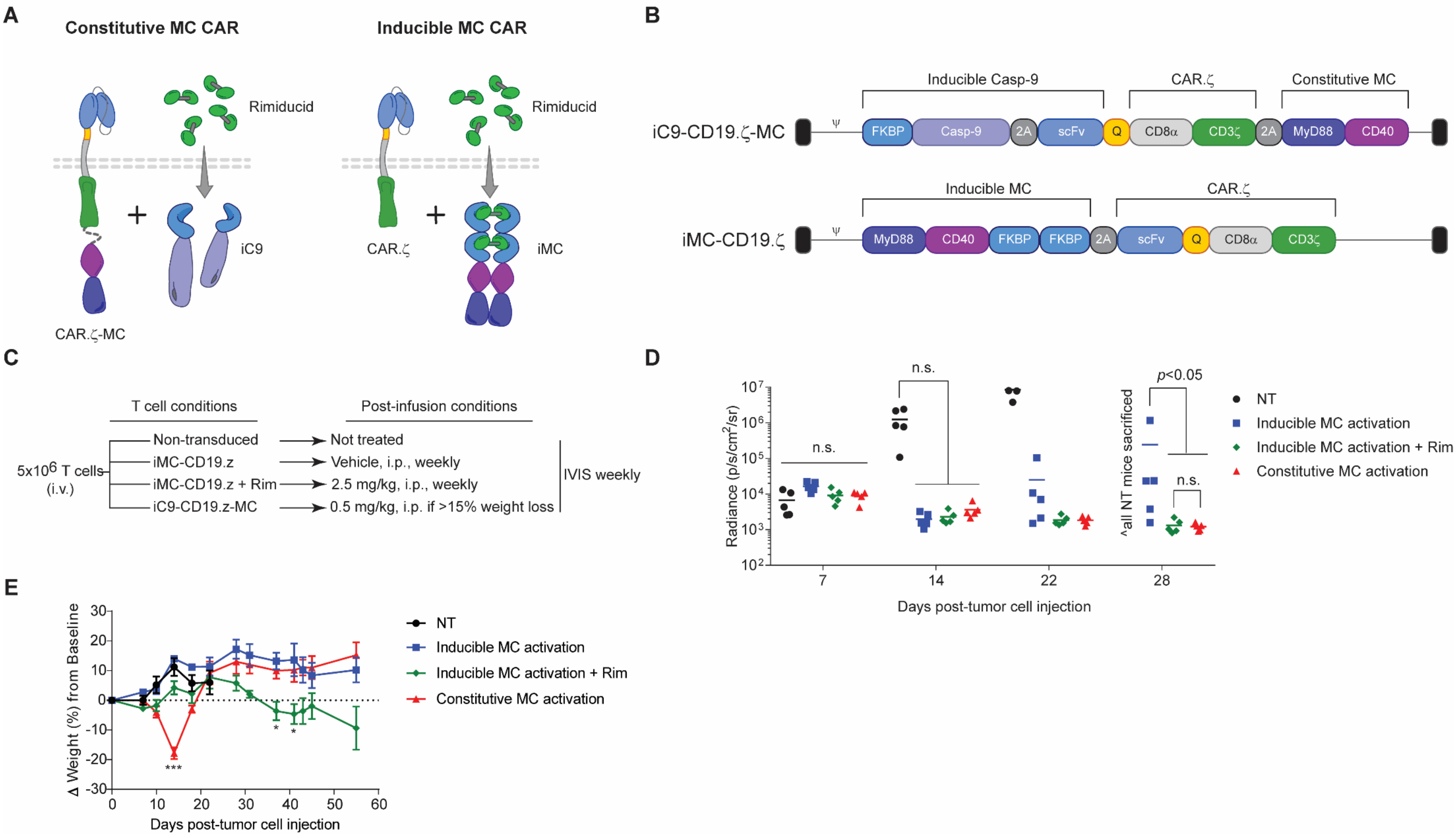

Supplementary Figure 4. High basal activity from constitutive MC expression shows similar anti-tumor activity to an inducible MC system against CD19⁺ tumor cells. A) Schematic comparing two CAR systems using constitutively active MC versus rimiducid-dependent MC dimerization to provide MC stimulation. **B)** Comparison of iC9-CD19.ζ-MC and iMC-CD19.ζ constructs. The details of iC9-CD19.ζ-MC have been described in Figure 2. iMC is comprised of two tandem FKBP12 binding domains cloned in frame with the MyD88 and CD40 signaling domains. The CD19-specific

CAR is identical to the iC9-CD19.ζ-MC CAR. **C**) NSG mice were injected with $5x10^5$ Raji-EGFPluc tumor cells via tail vein injection. After 4 days, mice received NT T cells or T cells transduced with either the iMC-CD19.ζ or iC9-CD19.ζ-MC retroviral vector at a single dose of $5x10^6$ cells per mouse. In one group that received iMC-CD19.ζ-modified T cells, the mice received weekly injections of rimiducid through an i.p. injection of 5 mg/kg starting on day +1 post-T cell injection. **D)** *In vivo* imaging was performed to measure tumor growth using luciferase bioluminescence. **E)** Weights were tracked as a percent of the initial baseline measurement for each animal. Statistical comparisons to Inducible MC activation. * represents a P-value <0.05, *** represents a P-value <0.005.

Supplementary Figure 5

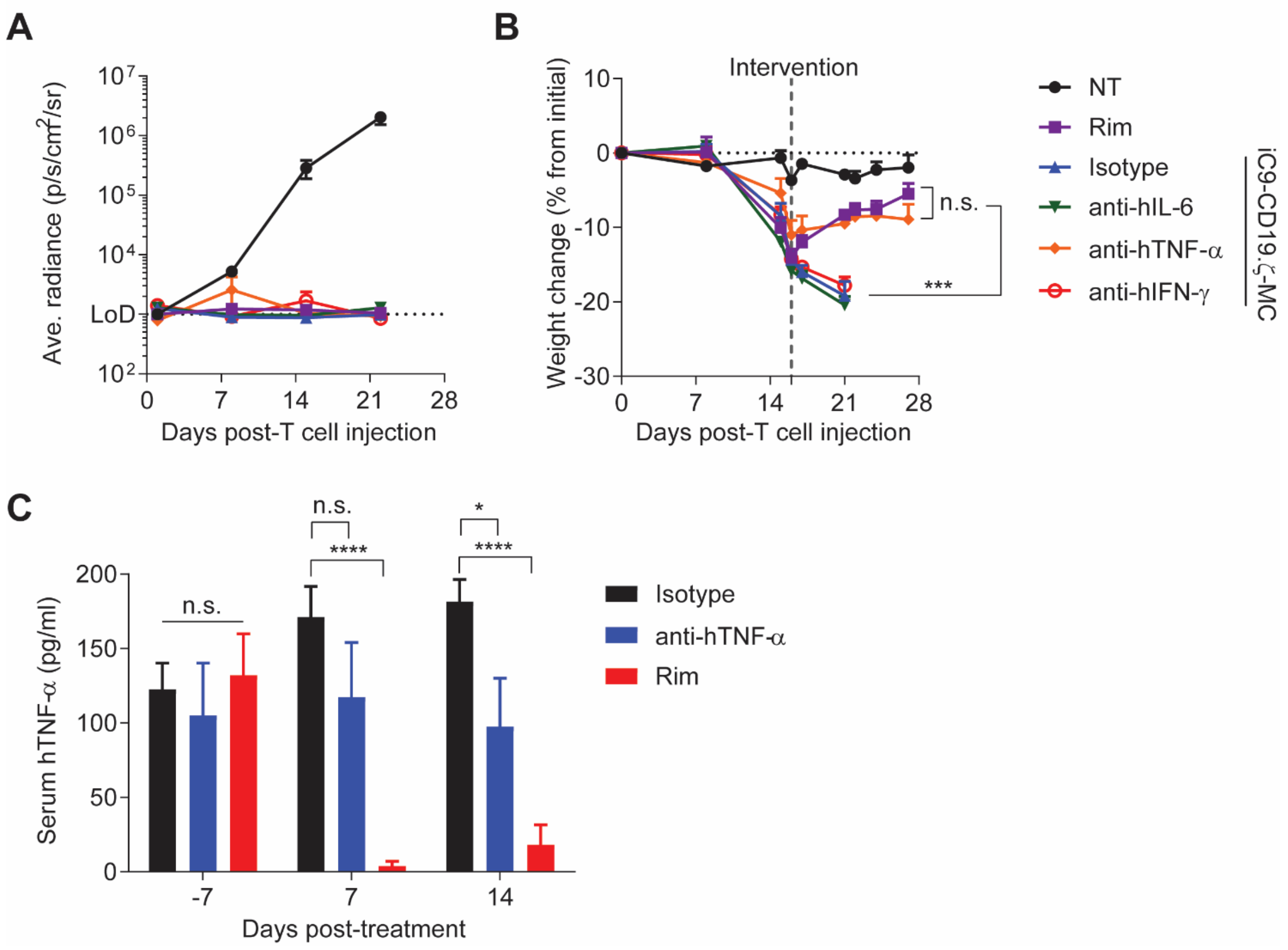

Supplementary Figure 5. CAR-T cell cytokine production responsible for murine toxicity. NSG mice engrafted with $CD19^{+}$ Raji-EGFPluc tumor cells were treated with $5x10^{6}$ non-transduced (NT) or iC9-CD19.ζ-MC-modified T cells. Mice receiving CAR-T cells were subsequently treated by twice weekly i.p. Injections of neutralizing antibodies to hIFN-γ, hIL-6 or hTNF-α, or a control non-specific isotype antibody after >10% weight loss was observed (day 15). As a control, one group was given 5 mg/kg rimiducid to resolve toxicity. **A)** Tumor growth was measured by bioluminescent imaging (BLI) and **B)** CAR-dependent toxicity by measuring weight loss. **C)** Serum concentration of hTNF-α was measured on days -7, 7, and 14 post-administration of neutralizing antibody cycle.

Supplementary Figure 6

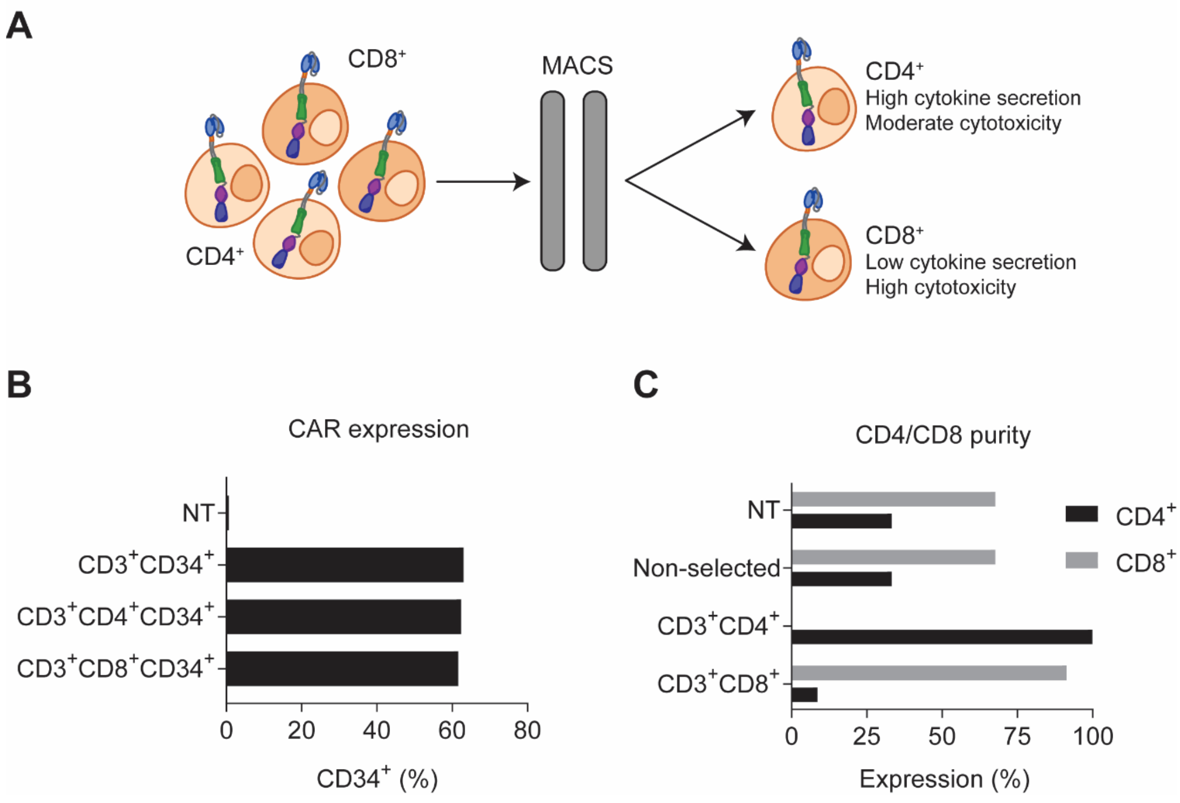

Supplementary Figure 6. T cell subset selection for minimizing cytokine production from T cells engineered with iC9-CAR.z-MC vectors. A) Transduced T cells forming a bulk population containing both CD4+ (high cytokine producers) and CD8+ (low cytokine production) were purified for either CD4 or CD8 expression using MACS columns. **B)** CAR expression of non-transduced (NT), unselected or CD4 and CD8-selected CAR-T cells. **C)** Purity of unselected and selected CAR-T cells.
